# Social media to supplement point-of-care ultrasound courses: the “sandwich e-learning” approach. A randomized trial

**DOI:** 10.1186/s13089-016-0037-9

**Published:** 2016-03-12

**Authors:** Dorothea Hempel, Stephanie Haunhorst, Sivajini Sinnathurai, Armin Seibel, Florian Recker, Frank Heringer, Guido Michels, Raoul Breitkreutz

**Affiliations:** Department of Cardiology, Angiology and Intensive Care, University Medical Center Mainz, Mainz, Germany; Department of Anesthesiology and Intensive Care Medicine, Kliniken Maria Hilf, Moenchengladbach, Germany; Department of Anesthesiology, Intensive Care and Emergency Medicine, Diakonie Klinikum Jung Stilling, Siegen, Germany; Ultrasound Network in Acute and Critical Care, SonoABCD, Frankfurt am Main, Germany; Sono4Students, University Hospital Bonn, Bonn, Germany; FINeST Frankfurt Institute of Interdisciplinary Emergency Medicine and und Simulation Training, Johann Wolfgang Goethe University, Frankfurt am Main, Germany; Department III of Internal Medicine, Heartcenter, University Hospital of Cologne, Cologne, Germany; Emergency Department, Hospital of the City of Frankfurt (Höchst), Gotenstr. 6-8, 65929 Frankfurt am Main, Germany

## Abstract

**Background:**

Point-of-care ultrasound (POC-US) is gaining importance in almost all specialties. E-learning has been used to teach theoretical knowledge and pattern recognition. As social media are universally available, they can be utilized for educational purposes. We wanted to evaluate the utility of the *sandwich e*-*learning* approach defined as a pre-course e-learning and a post-course learning activity using Facebook after a one-day point-of-care ultrasound (POC-US) course and its effect on the retention of knowledge.

**Methods:**

A total of 62 medial students were recruited for this study and randomly assigned to one of four groups. All groups received an identical hands-on training and performed several tests during the study period. The hands-on training was performed in groups of five students per instructor with the students scanning each other. Group 1 had access to pre-course e-learning, but not to post-course e-learning. Instead of a pre-course e-learning, group 2 listened to presentations at the day of the course (classroom teaching) and had access to the post-course learning activity using Facebook. Group 3 had access to both pre- and post-course e-learning (*sandwich e*-*learning)* activities, while group 4 listened classroom presentations only (classroom teaching only). Therefore only groups 2 and 3 had access to post-course learning via Facebook by joining a secured group. Posts containing ultrasound pictures and videos were published to this group. The students were asked to “like” the posts to monitor attendance. Knowledge retention was assessed 6 weeks after the course.

**Results:**

After 6 weeks, group 3 achieved comparable results when compared to group 2 (82.2 % + −8.2 vs. 84.3 + −8.02) (*p* = 0.3). Students who participated in the post-course activity were more satisfied with the overall course than students without post-course learning (5.5 vs. 5.3 on a range from 1 to 6).

**Conclusions:**

In this study, the *sandwich e*-*learning* approach led to equal rates of knowledge retention compared to classroom lectures and post-course learning. Students appreciate new media for learning experiences and are more satisfied with their learning activity. The sandwich e-learning can be used to maximize hands-on training during courses.

**Electronic supplementary material:**

The online version of this article (doi:10.1186/s13089-016-0037-9) contains supplementary material, which is available to authorized users.

## Background

Social Media such as Facebook, Twitter, and YouTube are changing the way medical educators are teaching today. A significant percentage of students already use online resources and social media as means of self-directed learning [1–3]. The most popular and widely used network is Facebook with over 1.32 billion registered users, 945,000,000 of them using mobile devices [4]. Facebook allows users to communicate through chats or via private messages on an individual basis or in defined groups. Students already use Facebook for educational purposes on a daily basis [5, 6].

Ultrasound is an increasingly important diagnostic tool and is amenable to online learning. Facebook offers a broad variety of communication tools (messages, videos via walls, posts, etc.) that distinguish it from other media such as Twitter that are limited to 140 characters. A challenge in medical education has always been to teach not only for the moment but to achieve retention of knowledge over time. E-learning using social media and other forms such as password-secured platforms are increasingly being used by medical schools and in post-graduate education. The advantage of e-learning is that the learner can autonomously define time, pace, and location of the activity. As the use of social media is increasing, recommendations for the integration into educational programs have been published [7–9].

Hands-on training still is an integral part of every ultrasound course and is expected by the participants [10]. The time spent on this activity should be maximized.

In this study, we intended to assess the value of a pre-course e-learning combined with a post-course activity using Facebook, a concept we defined as *sandwich e*-*learning*. Our primary goal was to analyze the effect of this combined pre- and post-course e-learning (*sandwich e*-*learning*) activities on the retention of knowledge compared to standard lectures and post-course learning; the secondary aim was to evaluate the utility of post-course activity using social media.

## Methods

We developed a one-day course based on emergency ultrasound courses offered by the DEGUM (German Society for Ultrasound in Medicine) and the German Society of Anesthesiology and Intensive care (Deutsche Gesellschaft für Anästhesie und Intensivmedizin; DGAI; curriculum of AFS module 5). The curriculae incorporate the E-FAST exam (extended focused assessment with sonography in trauma) and clinical applications of thoracic ultrasound such as pleural effusion, B-lines, and pulmonary edema, pneumothorax, and consolidations [11].

### Participants

We recruited medical students without prior ultrasound experience (3rd year and higher) from two medical schools in Germany (University of Frankfurt and University of Bonn). Participation in the course was not required for completing the curriculum at university and had no influence on the progress or grading of their medical studies. For participating in the study, all students received an expense allowance of 50 Euros and a compensation for their traveling expenses. All were given ultrasound pocket-sized cards (www.SonoABCD.org) and a textbook summarizing the content of AFS Module 5 after the end of the study. The cards are pocket-sized cards summarizing scanning techniques, physiologic, and pathologic findings. We developed five different cards covering POC-US as an introduction, the FAST exam, thoracic ultrasound, the FEEL exam (focused echocardiographic evaluation in life support [12]), and deep vein thrombosis. Every student received the FAST and the thoracic ultrasound pocket cards. The textbook handed out to the students was published by two of the authors (RB and AS) as complementary material to the certified ultrasound courses. To motivate the students, the three top scorers were invited to participate in another ultrasound course free of cost. All participants gave written consent for their data being used for research purposes and publication; an approval by the ethics committee was waived.

### Study design

**Fig. 1:**
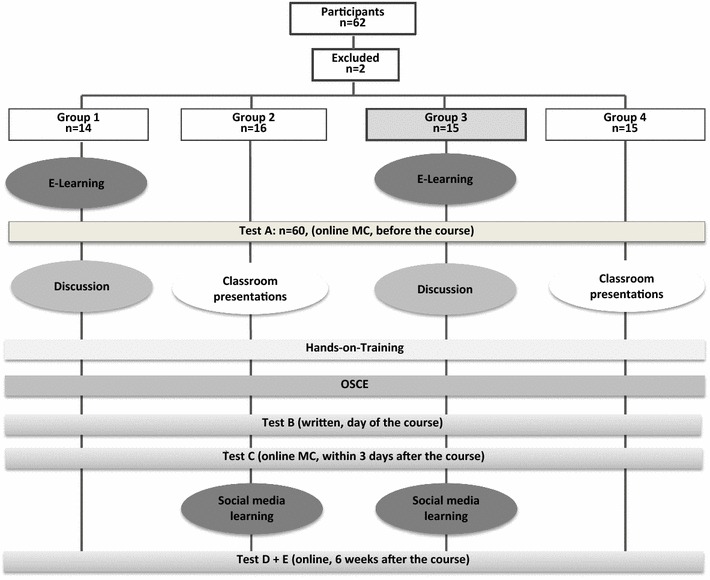
Study design: Group 3 underwent the *sandwich e*-*learning* approach

The study was designed as a randomized, controlled, parallel group study. Students were randomized into four groups (G 1–4) (Fig. 1).

All students were required to take a multiple choice test (Test A) consisting of 20 questions including multiple choice questions (MC) and drag-and-drop tasks during the 4 weeks prior to the course.

The pre-course e-learning consisted of 14 case-based screencasts with a maximum duration of 5 min. The content of the screencasts is listed in Table 1. The screencasts, defined as digital recordings of a computer screen output enhanced with audio narration [13], were produced by seven experienced physicians using a standard template. All physicians were certified POCUS instructors and were taught how to use the free online program screencast-o-matic (www.screencast-o-matic.com) for production of the screencasts. Every presentation included a standard structure of the following slides: *title, presentation of the case/problem, ultrasound images or clips, interpretation/progress of the case, key messages, conclusion, questions*, and *title again* [14]. Screencasts were uploaded as an e-learning curriculum onto a password-secured platform.

**Table 1:** Contents of the screencasts and the lectures

Screencast no.	Case vignette	Content	Corresponding lecture (no.)
1	Fall from 3 m height	FAST algorithm and probe positioning	7
2	Motorcycle accident	Positive FAST: free fluid in right upper quadrant	9
3	Bicycle accident	Positive FAST: free fluid in left upper quadrant	9
4	Syncope in a pregnant patient	Positive FAST: free fluid in pelvis	9
5	Resuscitation after thoracic surgery	Pericardial effusion	8
6	Dyspnea after central line placement	Physiologic artifacts in lung ultrasound: lung sliding, A-lines, lung pulse	1
7	Weaning failure and intubation	Pleural effusion, compression atelectasis, B-lines	4
8	Dyspnea I	Sign of pulmonary fluid: multiple B-lines	5
9	Dyspnea II	B-lines: Pathophysiology and scanning technique	2
10	Dyspnea III	Pleural effusion, ultrasound-guided thoracentesis	4
11	Dyspnea IV	Consolidations, aerobronchogram, pneumonia	5
12	Car crash	Rib fracture, exclusion of pneumothorax	6
13	Tracheostomy	Ultrasound of the trachea, tracheotomy under ultrasound-guidance	3
14	Respiratory distress after subclavian line placement	Lung ultrasound signs of pneumothorax	6

G 1 and G 3 were given access to a case-based e-learning curriculum 4 weeks prior to the course and participated in a discussion (60 min) on the day of the course. The discussion was moderated by two tutors and intended to clarify all issues that arose during the e-learning.

G 2 and 4 (classroom groups) had no access to the pre-course e-learning and listened to standardized classroom-based presentations on the day of the course. The classroom teaching consisted of nine lectures lasting 15 min each, covering identical content as that for the pre-course e-learning (Table 1).

All groups participated in a hands-on training (HT) identical in structure and content (180 min in total). HT was performed in small groups of five students rotating through six stations with the students scanning each other under supervision of an experienced instructor who was not compensated for the activity (stations 1–3: thoracic ultrasound; stations 4–6: FAST).

After the HT, every participant performed a standardized objective clinical skills examination (OSCE) with the maximum score of 50. The test required the students to perform a defined exam, i.e., FAST, and name structures displayed. The instructors evaluated the tasks as right (1 point) or wrong (0 points). With a total of 50 tasks, the maximum score that could be achieved was 50.

**Fig. 2:**
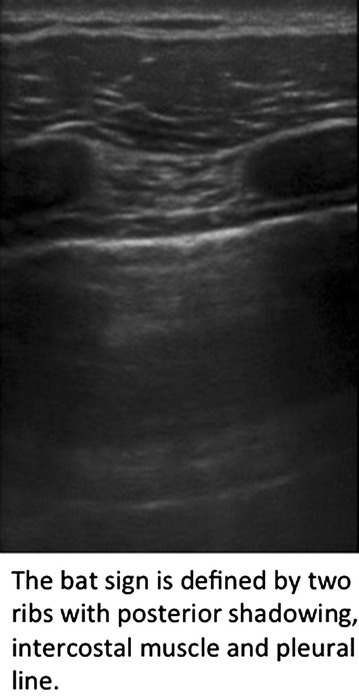
Example of a post with a picture and a short text

After the course, G 2 and G 3 were invited to join a password-protected group in the social network Facebook. Every weekday, a Facebook post was posted to the group for a total of 6 weeks (30 tweets). Each post had a maximum word count of 140 characters; 17 out of 30 included an ultrasound video or image (Fig. 2). To monitor attendance, students were instructed to like each of the posts. The original posts can be found in the Additional file 1: Table S1.

All students performed a MC test (test B) on the day of the course; an online MC test within 3 days after the course (test C); and an online MC test 6 weeks after (test D). Another test (test E) consisted of 20 questions including pictures and videos showing physiologic or pathologic findings. These questions had to be answered in free text and were intended to assess the pattern/image-recognition ability of the students.

Students were also asked to answer a questionaire after the day of the course and after completion of the study protocol grading items from one (poor) to six (very good). The survey covered every phase of the study (pre-course, classroom lectures, HT, post-course, tests, etc.) and asked about their satisfaction with organization, layout, content, and applicability.

### Statistical analysis

Statistical analysis was performed using GraphPad Prism version 6.0 (GraphPad software, Inc. La Jolla, CA 92037, USA). Test results were analyzed and presented in % score with 25 and 75 % percentiles.

The Mann–Whitney-U test was used for group comparison. We chose a significance level of *α* = 5 %. The primary outcome was the performance in the MC tests and the OSCE; therefore, a Bonferroni–Holm correction for multiple testing was applied, and power calculation was performed for *α* = 2.5 %.

## Results and discussion

**Fig. 3:**
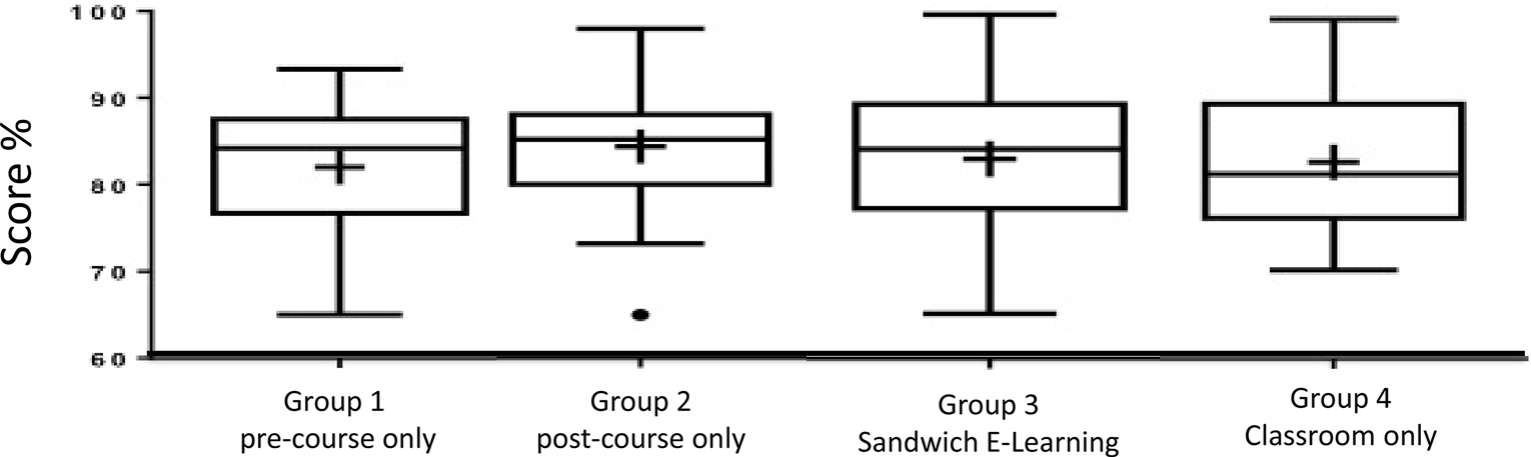
Results of test D: no difference in performance between the groups

A total of 62 students were recruited from the universities of Bonn and Frankfurt. Two students did not attend the course, while three students did not complete test C, leaving 57 students for this statistical analysis. When comparing group 3 (sandwich e-learning) with the other groups (post-course learning alone, pre-course learning only, and classroom-based), no significant differences were found both in test D and test E (*p* = 0.3; *p* = 0.5) (Fig. 3). Groups 1 and 3 performed significantly better in test A compared to groups 2 and 4 (*p* < 0.05). Results of tests B and C did not show any significant difference. The results and a detailed discussion of tests A, B, and C have previously been published elsewhere [14].

The evaluation by the participants showed that the post-course learning raised the satisfaction as G 2 and 3 evaluated the course with a mean score of 5.5 (95 % CI 5.7–5.3) compared to a mean score of 5.3 (95 % CI 5.5–5.1) given by G 1 and 4 (*p* < 0.05).

81.8 % of the participants evaluated the post-course activity as good or very good with an adequate amount of time spent on this activity. 60.6 % of students felt their increase in knowledge was good; this was only their subjective judgement.

In this study, we designed and applied the *sandwich e*-*learning* technique, a novel approach defined as a learning pathway including a pre-course e-learning curriculum, a hands-on training (HT) session, and a post-course activity using social media after the course.

Teaching POC-US ultrasound has to incorporate different aspects of learning. They are the sensor–motor component of moving the ultrasound probe, the skill of pattern recognition, and the skill of incorporation of the findings into the clinical setting. Pattern-recognition training can be imparted utilizing e-learning. The integration into the clinical context can be partially trained unsing e-learning by adding short case vignettes. Description, for example, of the history of present illness, vital signs, etc., in combination with an ultrasound picture or video, can instead be used in the training for the integration of the ultrasound findings into the clinical context. The motor skills of probe placement on the patient can most effectively be learned via hands-on training [15]. Many studies have evaluated educational methods for teaching procedural skills [16, 17]. Hands-on training is very staff-intensive as the number of students trained by one teacher should be as small as possible to allow the individual student enough time. Many physicians and students learn the basics of POC-US in focused courses lasting only one to 2 days [18, 19]. Therefore, hands-on time during these courses should be maximized.

E-learning both traditional methods such as online curricula and new methods utilizing social media such as Facebook can be used to teach theoretical knowledge and pattern recognition [5, 6]. The advantages of e-learning are flexibility regarding space, time, and duration [20], and in contrast to generic courses, the form of a self-organized learning pathway over time which can enhance retention of knowledge. It is important to point out that every educational activity using social media needs to be rigorous regarding the appropriate use and privacy policies that apply [7].

## Conclusions

In our study, students trained with the *sandwich e*-*learning* approach achieved similar test results after 6 weeks compared to the groups with traditional teaching methods such as classroom lectures. Therefore, we argue that e-learning might be an alternative to classroom lectures as it offers the possibility to maximize the time spent on hands-on training and seems to be a feasible method of learning in addition to hands-on training for POC-US.

## Limitations

In this study, we only analyzed the effect of the sandwich e-learning approach on a limited number of medical students; further research is needed applying this approach to postgraduate training where the use of social media is not as common, especially regarding ultrasound education. We assume that all participating students had a high motivation, but we did not perform a motivational analysis. The results of the online tests are limited by the fact that students could have used other resources while completing the tests. As all groups would have this possibility, this does not contradict the results and conclusion of our study. Our study is further limited by the fact that we only assessed the retention of knowledge after 6 weeks. Further study comparing different models of sandwich e-learning, i.e., social media-based pre-course and post-course versus a structured curriculum-based pre-course and post-course, is needed to explore the efficacy of this technique. More detailed investigation is also needed to ascertain if knowledge is retained over longer periods of time and when it is applied in clinical practice.

## Additional file

10.1186/s13089-016-0037-9 The posts that were sent to the Facebook group.
