# Supplementary material for: Social media to supplement point-of-care ultrasound courses: the “sandwich e-learning” approach. A randomized trial
Source: Crit Ultrasound J. 2016 Mar 12;8:3. doi: 10.1186/s13089-016-0037-9 (PMC4788673; doi:10.1186/s13089-016-0037-9)
Supplement: Supplementary file 1 — 10.1186/s13089-016-0037-9 The posts that were sent to the Facebook group. [file 13089_2016_37_MOESM1_ESM.docx]

Additional file 1: Table S1

| **Day** | **Image/clip** | **Content** |
| --- | --- | --- |
| 2 | 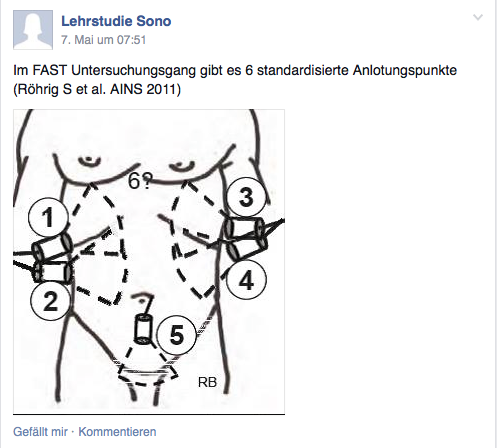 | The FAST exam consists of 6 standardized probe positions (Röhrig S et al. AINS 2011) |
| 3 | 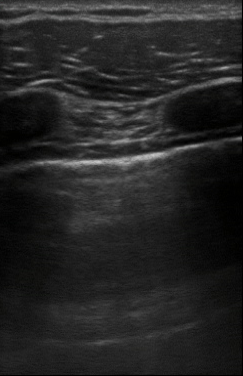 | The bat sign is defined by two ribs with shadows,  intercostal muscles and the pleural line |
| 4 | 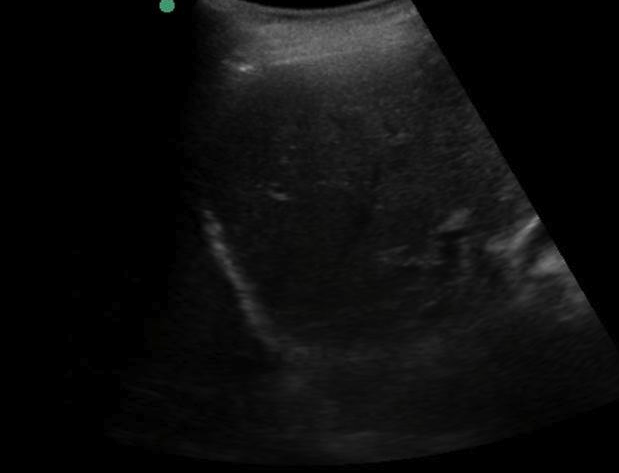 | FAST 1: look for pleural effusion and  free fluid below the diaphragm on the right side |
| 5 |  | B-lines arise from the pleural line and reach the lower end of the screen |
| 6 | 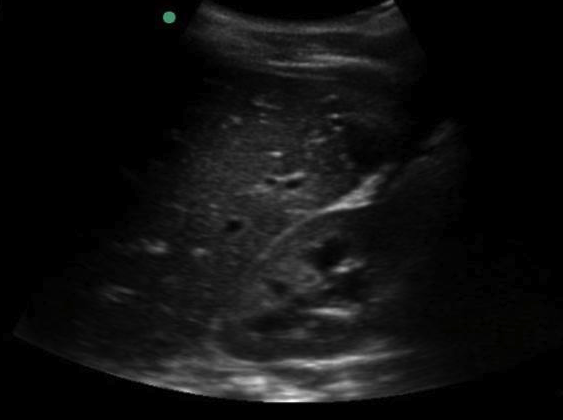 | FAST 2, Morison-Pouch: diagnose or exclude free fluid between liver and kidney |
| 9 |  | Movement of the pleura is seen as lung sliding |
| 10 | 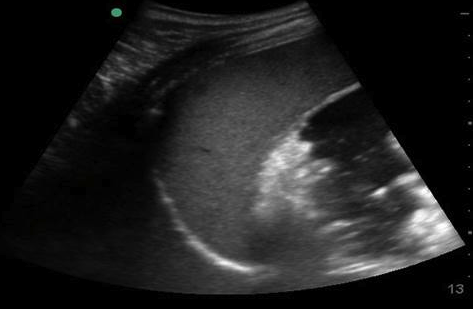 | FAST 3: look for pleural effusion and free fluid  below the diaphragm on the left side |
| 11 |  | Lungsliding in M-mode🡪 „seashore sign“ |
| 12 |  | FAST 4: look for free fluid between spleen and kidney |
| 13 | 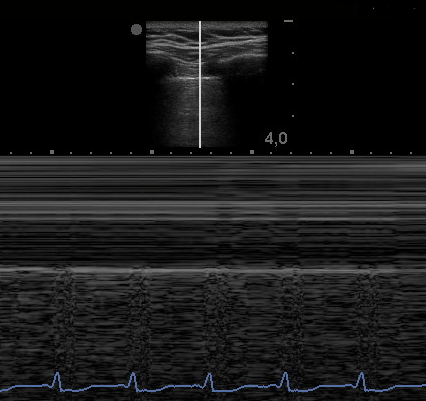 | The movement oft he heart is transferred via lung and pleura to the surface and can be seen as lung pulse |
| 16 | 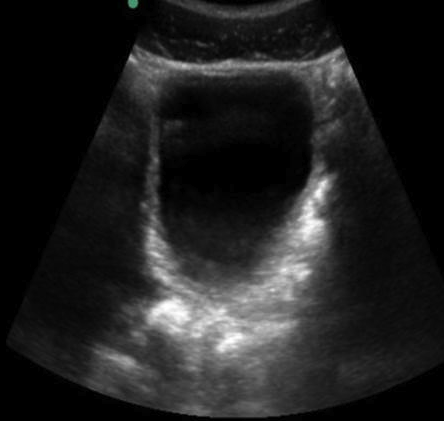 | FAST 5: free fluid behind and around the bladder |
| 17 | 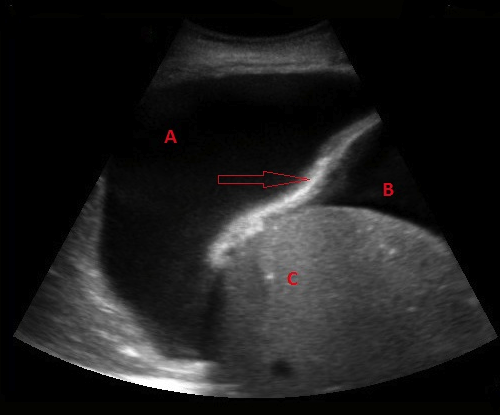 | Can you name the structures labeled A-C?  Which structure is marked by the arrow? |
| 18 | 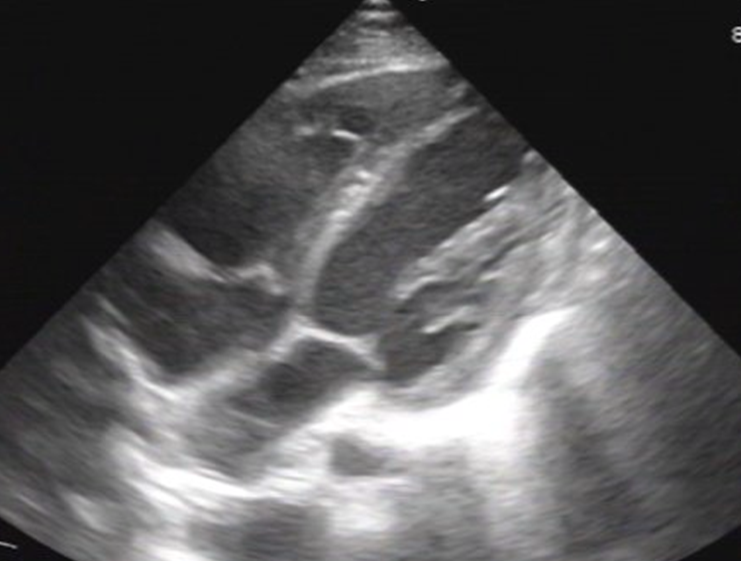 | FAST 6: subcostal view; exclude a pericardial effusion |
| 19 |  | Signs of a pneumothorax are: lung point, absence of lung sliding, b-lines and lung pulse |
| 20 | 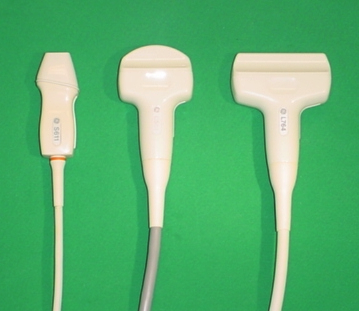 | Which probe would you use for the FAST? What depth is needed? |
| 23 |  | The lung point is the interface between the area where the pleural layers are not attached and where they are attached. |
| 24 |  | FAST is integrated into the ATLS-protocol during the primary survey: How long does a FAST exam take? |
| 25 |  | By showing the lung pulse a pneumothorax can be excluded |
| 26 |  | FAST can help establishing transport and treatment priorities during a mass casualty incident |
| 27 |  | B-lines represent fluid-filled lung tissue |
| 30 | 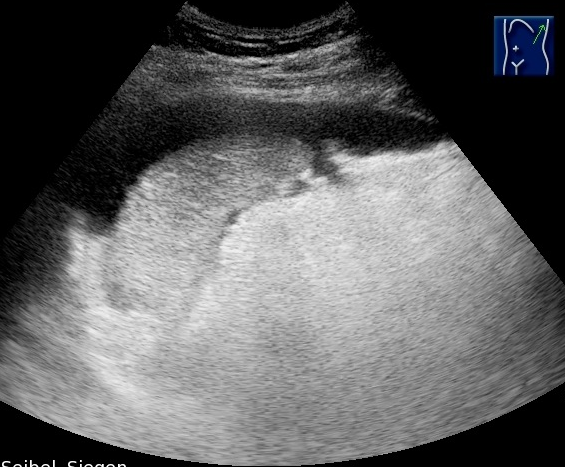 | Trauma: Would you bring a patient to the OR relying solely on an ultrasound? |
| 31 | 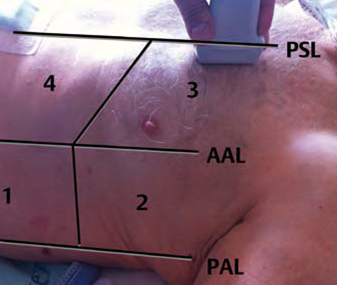 | Each hemi-thorax is divided into four quadrants |
| 32 |  | In case of an abdominal injury in-hospital mortality increases every 3 minutes by 1% |
| 33 |  | Two air-mucosa interfaces after intubation: sign for esophageal intubation, you can make the diagnosis without ventilating the patient! |
| 34 |  | Pre-hospital evidence for intra-abdominal bleeding: sensitivity 93%, specificity 99%  (Walcher F. et al., Brit J Surg 2006) |
| 37 |  | A-lines are reverberation artifacts of the pleura |
| 38 |  | E-FAST: combining the FAST exam with lung ultrasound looking for pneumothorax. Probe position:  medio-clavicular line, 3rd/4th Intercostal space |
| 39 | 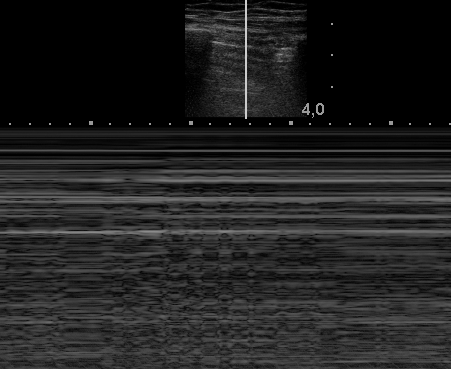 | In case of a pneumothorax: due to air in the pleural space no lung sliding is visible on M-mode = stratosphere sign. |
| 40 |  | Pitfalls: hemodynamic instability in the presence of a negative FAST: beware of bleeding (cranial, retroperitoneal, pelvis, hip) |
| 41 |  | Pulmonary edema is characterized by multiple and bilateral  B-lines |
